# Typhoid in Laos: An 18-Year Perspective

**DOI:** 10.4269/ajtmh.19-0637

**Published:** 2020-01-27

**Authors:** Tamalee Roberts, Sayaphet Rattanavong, Koukeo Phommasone, Vilada Chansamouth, Viengmon Davong, Valy Keoluangkhot, Sitthivone Hongsakhone, Naly Bounsavath, Mayfong Mayxay, Manivanh Vongsouvath, David A. B. Dance, Paul N. Newton

**Affiliations:** 1 Lao-Oxford-Mahosot Hospital-Wellcome Trust Research Unit, Microbiology Laboratory, Mahosot Hospital, Vientiane, Lao People’s Democratic Republic;; 2 Nuffield Department of Medicine, Centre for Tropical Medicine and Global Health, University of Oxford, Oxford, United Kingdom;; 3 Adult Infectious Diseases Ward, Mahosot Hospital, Vientiane, Lao People’s Democratic Republic;; 4 Oudomxay Provincial Health Department, Oudomxay, Lao People’s Democratic Republic;; 5 Huaphan Provincial Health Department, Huaphan, Lao People’s Democratic Republic;; 6 Institute of Research and Education Development, University of Health Sciences, Vientiane, Lao People’s Democratic Republic;; 7 Faculty of Infectious and Tropical Diseases, London School of Hygiene and Tropical Medicine, London, United Kingdom

## Abstract

Although typhoid is endemic to Southeast Asia, very little is known about the disease in Laos. Typhoid vaccination is not included in the national immunization program. Although sanitation has improved, one million people still do not have access to basic clean water sources. We describe the epidemiology and antimicrobial susceptibility patterns of *Salmonella enterica* serovar Typhi (*S*. Typhi) infection in Laos based on isolates accrued over 18 years at Mahosot Hospital, Vientiane. All blood cultures collected from patients presenting with fever submitted to the Microbiology Laboratory at Mahosot Hospital (February 2000–December 2018) were included. This included patients from Vientiane and four provincial hospitals and one typhoid outbreak investigation. A total of 913 (1.5%) of 60,384 blood cultures were positive for *S.* Typhi. The majority of isolates with data available (712/898, 79.3%) were susceptible to all antibiotics tested, with 59 (6.5%) multidrug-resistant (MDR) isolates, mostly from one outbreak. Of 854 isolates, 12 (1.4%) were fluoroquinolone resistant. Patient admissions peaked between March and June at the end of the dry season. Although there are key limitations, these data give the first detailed epidemiological evidence of typhoid in Laos. However, estimates will be greatly influenced by access to blood culture services and health-seeking behavior. Although typhoid multidrug resistance and fluoroquinolone resistance are not currently major issues in Laos, continued surveillance and improved antibiotic stewardship are necessary to forestall worsening of the situation. Cost-effectiveness analysis is needed to inform decisions regarding typhoid vaccine introduction.

## INTRODUCTION

Typhoid, caused by *Salmonella enterica* serovar Typhi (*S*. Typhi), is transmitted through contaminated food and water and is estimated to cause > 10 million illnesses and 128,000 deaths annually worldwide.^1,2^ *Salmonella enterica* serovar Typhi is an important cause of community-acquired bacteremia in South and Southeast Asia. Thailand reported a decrease in cases from 8.6 cases/100,000 people in 2003 to 3/100,000 people in 2014,^3^ and Vietnam has reported a continued decline from an estimated incidence of 14.7/100,000 people in 2003.^4^ Immunization programs were started in Thailand in the 1970s and Vietnam in the 1990s, which may have led to these decreases in case burden.^3,4^ The role that water, sanitation, and hygiene initiatives and economic growth have also had in this decline is difficult to judge, although they have probably played a major role. The WHO has long recommended immunization for people living in endemic areas and has recently advised the use of a new prequalified typhoid conjugate vaccine.^5^

There is a high frequency of multidrug-resistant (MDR) *S*. Typhi in South and Southeast Asia. Multidrug-resistant *S.* Typhi is defined as resistant to first-line antibiotics ampicillin, chloramphenicol, and trimethoprim–sulfamethoxazole (co-trimoxazole). In Southeast Asia, MDR *S.* Typhi was first reported in Vietnam in 1992 and the first quinolone resistance in 1993.^6^ An increase in the frequency of resistance to cephalosporins, the current main parenteral treatment of typhoid fever, has also been observed in South and Southeast Asia.^7^ These changes in resistance patterns make control of *S.* Typhi even more important. As *S.* Typhi is a pathogen restricted to humans, it may be possible to eliminate it with appropriate vaccination and sanitation interventions.

Although *S*. Typhi is endemic in Laos and was first described clinically in 1909 and from blood culture in 1995 (Phetsouvanh et al.,^8^ Clarkson et al. submitted), there are relatively few published data about typhoid in Laos. The first report of the causes of bacteremia in Laos between 2000 and 2004 included 246 (50.9% of clinically significant bacteremias) *S.* Typhi isolates from 4,512 blood culture bottle pairs.^9^ A prospective study of the use of the rapid diagnostic test (RDT) (using OneStep *Salmonella* Typhi Ag Rapid Detection Kit; Standard Diagnostics, Kyonggi-do, South Korea) in Mahosot Hospital between September 2010 and December 2011 found *S.* Typhi in 30 (0.99%) of 3,028 blood cultures.^10^ Subsequently, it was shown that *gyrA* mutations could be detected in RDT-derived DNA to predict fluoroquinolone resistance in *S.* Typhi.^11^

In a parallel article, we used data from sentinel surveillance from Mahosot Hospital over a 2-year period combined with a healthcare utilization survey in the hospital’s main catchment area of Vientiane to develop an estimate of enteric fever incidence in the community. This study found an estimated annual incidence of typhoid of 4.7 per 100,000 persons.^12^ A clinical trial carried out in Vientiane between 2001 and 2003 showed that 3 days of ofloxacin was more efficacious than 14 days of chloramphenicol for uncomplicated inpatient typhoid treatment.^13^ The latest Lao National Treatment Guidelines follow this recommendation.^14^

There, thus, remains very limited information on the epidemiology and burden of *S*. Typhi infection in Laos. Vaccination is recommended for “most visitors” to Laos by some travel medicine expert systems, for example, the U.S. CDC and the United Kingdom National Travel Health Network and Centre.^15,16^ However, immunization against typhoid is not currently included in the Lao national program, but as this is now being considered, we reviewed the clinical, microbiological, and epidemiological data relating to isolates of *S.* Typhi obtained over the past 18 years at Mahosot Hospital, Vientiane.

## METHODS

### Site description.

Lao PDR (Laos) is a land-linked country in Southeast Asia, which had a reported population of 6,492,000 people in 2015.^17^ The climate is markedly seasonal with a wet season from late May to October, dry cool months from November to February, and dry hot months from March to May. Routine freely accessible blood culture diagnostic services are only currently available at central hospitals in Vientiane, the capital of Laos, where free blood culture for febrile patients was started at Mahosot Hospital in 2000.

### Blood samples.

All blood culture *S*. Typhi isolates identified by the Microbiology Laboratory at Mahosot Hospital (17°57′36.2″N, 102°36′43.3″E) between February 2000 and December 2018 were included. The majority of the blood cultures (698 specimens, 76.5%) were from patients admitted to Mahosot Hospital and other hospitals in Vientiane. In addition, isolates obtained during fever research studies (Xam Nua provincial hospital, Huaphan Province, from 2003 to 2006; Luang Namtha and Salavan provincial hospitals from 2008^18^; and Xieng Kuang provincial hospital from 2015) and an outbreak investigation (Oudomxay Provincial Hospital in 2004) are also included.

Adult blood culture bottles containing 20 mL tryptic hydrolysate of casein and soy peptone broth (TSB) plus 0.05% sodium polyanethol sulfonate were incubated in air at 37°C for 7 days (see Phetsouvanh et al.^9^ for more information). The bottles were examined daily for turbidity and were subcultured onto goat blood and chocolate agar if positive. “Blind” subcultures were also performed on days 1 and 7 post-inoculation. Bacteria were identified using standard microbiological techniques (API 20E, bioMérieux, Marcy L’Etoile, France) and were confirmed as *S*. Typhi by agglutination with O9, Hd, and Vi antisera (Becton Dickinson Laboratories, Franklin Lakes, NJ). Comprehensive antimicrobial susceptibility testing profiles were determined using disk diffusion on Mueller–Hinton agar (Oxoid, Basingstoke, UK) following the U.S. Clinical and Laboratory Standards Institute (CLSI) guidelines for the year the sample was collected and were all subsequently reinterpreted using the current CLSI guidelines (M100, 28th edition, January 2018). Isolates were tested against ampicillin, chloramphenicol, trimethoprim–sulfamethoxazole (co-trimoxazole), ceftriaxone, azithromycin, ciprofloxacin, and nalidixic acid. Where possible, isolates with missing antimicrobial susceptibility data, in particular ciprofloxacin for which testing started in 2013 and azithromycin for which interpretative criteria were only available from 2016, were retested in January 2019 to give a more complete set of antimicrobial susceptibility results. These isolates also had further confirmatory identification as *S.* Typhi, where possible, by Matrix assisted laser desorption/ionization time of flight mass spectrometry (MALDI-TOF MD) (bioMérieux). Laboratory records were also checked for discrepant results.

### Environmental factors.

Climate data for Vientiane Capital was exported from https://en.tutiempo.net/using the mean rainfall and temperature for each month. Information on water sources for villages was gathered from the 2005 and 2015 Lao census data^17,19^ (www.decide.la).

### Statistical analysis.

Typhoid hospital inpatient incidence of each district and province was standardized based on the number of reported infections diagnosed in patients reaching hospitals per 10,000 or 100,000 people, enumerated from the district and province population using the 2005 and 2015 Lao census data.^17,19^ With uncertainty about health-seeking behavior and access and with no data on community incidence, these data will be minimal estimates of the annual incidence of hospitalized typhoid patients and cannot reflect estimates of actual incidence in the community. Data were analyzed using Stata version 14 (StataCorp, College Station, TX). Mapping of cases was performed using Quantum GIS version 2.14.2 software. Relationships with water sources were compared using the chi-squared test.

### Ethics.

Ethical approval for the blood culture study was obtained from the Oxford Tropical Research Ethics Committee and from the National Ethics Committee for Health Research.

## RESULTS

**Figure 1.**
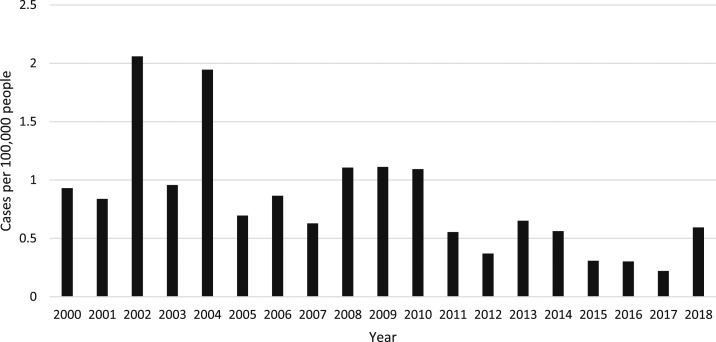
In-hospital incidence of *Salmonella enterica* serovar Typhi blood stream infections per 100,000 people in the community per year.

**Figure 2.**
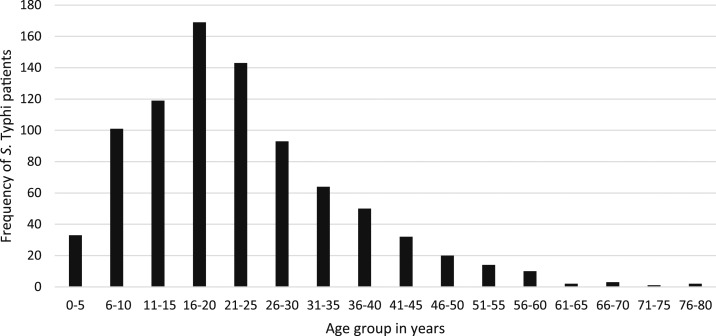
Frequency of *Salmonella enterica* serovar Typhi (*S*. Typhi) infections by age-group.

Of all blood culture sets collected during the 18 years, 1.5% (913/60,384) were positive for *S*. Typhi. Fifty-six (6.1%) patients were not admitted to the hospital, all from the outbreak in Oudomxay, but for clarity, all patients are regarded as inpatients in this analysis. The highest yearly in-hospital incidence was 2002, with 2.06 hospitalized patients per 100,000 people/year, with the most recent yearly in-hospital incidence of 0.59 cases per 100,000 people/year reported for 2018 (Figure 1). The year with the highest number of inpatients diagnosed was 2002 with 110 cases, followed by 2004 with 108 cases (Supplemental Figure 1). Typhoid inpatient numbers have decreased since 2010, but there has not been a steady trend. The median (range) age of patients was 21 (range 180 days–80 years) years, with the highest number of patients in the 16–20 years age-group (Figure 2, Table 1). Data from all individual patients were included in the study, although denominators vary for different parameters because of incomplete data. There was a small predominance of males (483/846, 57.1% with gender reported). All patients were reported to have had fever and 632 (78.4%) of 806 reported headache (Table 1). One hundred and seven (11.7%) patients reported having taken an antibiotic in the week before admission, most frequently ampicillin (17/79, 21.5% of those who could provide the antibiotic name). The admission diagnosis was recorded as typhoid in only 107/525 (20.4%) cases, whereas 108/525 (20.6%) were clinically diagnosed as having “rickettsial disease.” Patient outcome data were not available for most patients (684/913 patients, 74.9%) because of self-discharge and the lack of a formal follow-up system, but two patients were known to have died in hospital, 224 were known to be alive and improving at discharge, and one patient was discharged moribund. Information on ileal perforations was not available.

**Table 1:** Clinical symptoms associated with patients with *Salmonella enterica* serovar Typhi infection over 18 years

Variable	*n*
Patients culture positive for *S.* Typhi (%)	913/60,384 (1.5)
Median age (years) (range)	21 (180 days–80 years)
Female gender (%)	363 (39.8)
Mean duration of illness (days) (95% CI)	10.1 (8.9–11.2)
Mean admission temperature (°C) (95% CI)	38.7 (38.6–38.8)
Mean pulse (minutes) (95% CI)	97.3 (96.2–98.4)
Peripheral blood neutrophil (%), median (range)	67 (0.2–94)
Headache (%)	632/806 (78.4)
Rigors (%)	493/799 (61.7)
Nausea (%)	292/796 (36.7)
Vomiting (%)	223/808 (27.6)
Diarrhoea (%)	385/809 (47.6)
Myalgia (%)	358/522 (68.6)
Abdominal pain (%)	297/790 (37.6)

**Figure 3.**
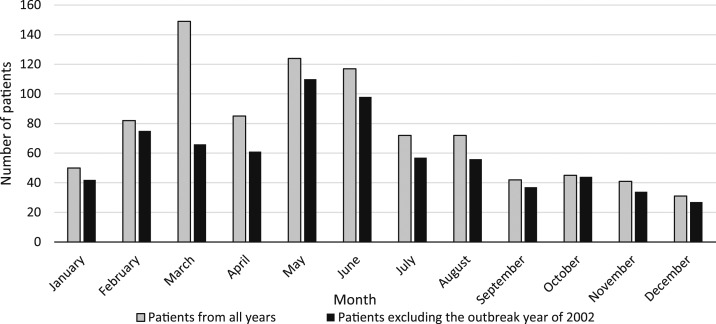
Total number of inpatients recorded with *Salmonella enterica* serovar Typhi blood stream infection per month for all years and total number of patients per month, excluding the outbreak year of 2002.

The number of cases peaked between the months of March and June (Figure 3), with the greatest number of cases reported in March 2002. Focusing on Vientiane Capital where the most complete weather data are available, there is a clear trend seen with increases in cases following the increase in temperature each year, with peaks usually occurring a month before the month of highest rainfall (Supplemental Figure 2).

**Figure 4.**
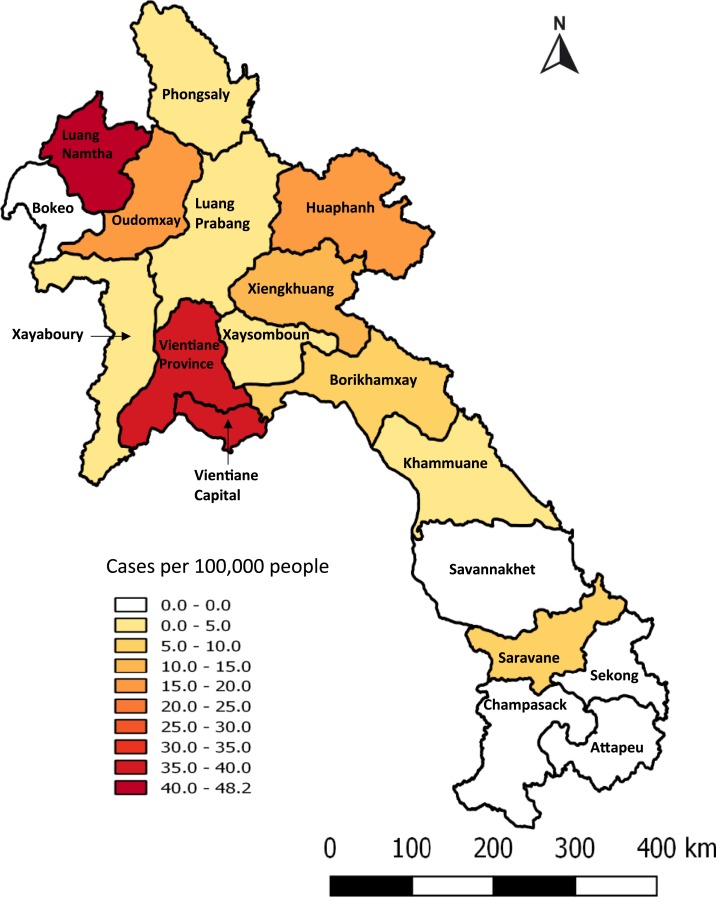
Total number of inpatients recorded with *Salmonella enterica* serovar Typhi bloodstream infection per 100,000 people per province from 2000 to 2018. This figure appears in color at www.ajtmh.org.

**Figure 5.**
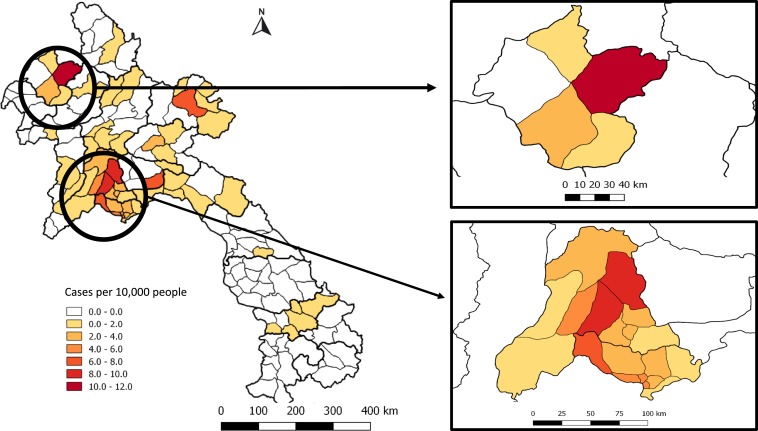
Number of inpatients recorded with *Salmonella enterica* serovar Typhi bloodstream infection per 10,000 people per district. Zoomed-in boxes represent Luang Namtha Province and Vientiane Capital and Vientiane Province. This figure appears in color at www.ajtmh.org.

Home location information was available for 737 (80.7%) of 913 patients, with the highest number coming from Vientiane Capital (295/737, 40.0%), followed by the neighboring Vientiane Province (155/737, 21.0%). However, when expressed as inpatients per 100,000 people over the 18-year period, the highest total incidence was in the northern province of Luang Namtha (48 hospitalized patients per 100,000 people from 2000 to 2018), mostly occurring between 2008 and 2014 (18 years total incidence; Figure 4, yearly incidence Supplemental Figure 3). Namtha district in Luang Namtha had the highest number of inpatients per 10,000 people with 11.8 cases/10,000 people from 2000 to 2018, followed by Vang Vieng and Hin Herb districts in Vientiane Province (8.6 and 8.2 cases/10,000 people from 2000 to 2018, respectively) (Figure 5). The highest number of inpatients from Vientiane Capital came from Sangthong district, with 7.5 cases/10,000 people from 2000 to 2018. In comparison, the highest proportions of positive blood cultures collected were from Oudomxay and Huaphan provinces. However, the data from these provinces are likely to be skewed as they largely came from investigations of suspected typhoid outbreaks (Supplemental Figure 4). Similarly, at a district level, the districts with the highest percentage of positive cultures were mainly from those in which less overall numbers of specimens were collected (Supplemental Figure 5).

### Antibiotic susceptibility data.

**Figure 6.**
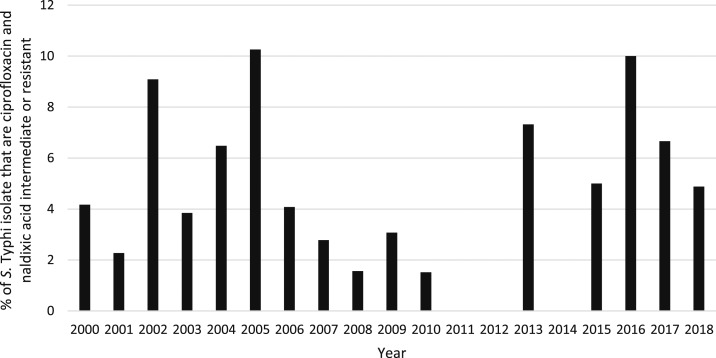
Percentage of *Salmonella enterica* serovar Typhi (*S.* Typhi) isolates that are ciprofloxacin and nalidixic acid intermediate or resistant per year.

**Figure 7.**
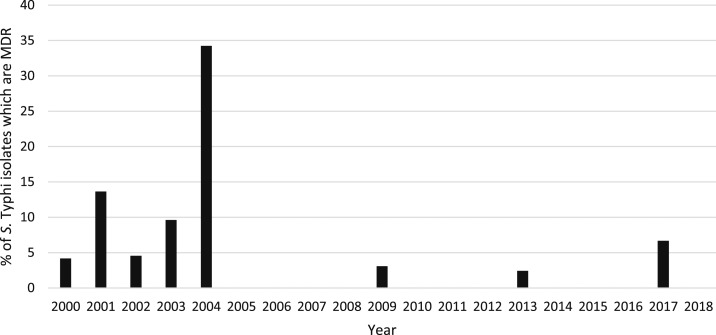
Percentage of *Salmonella enterica* serovar Typhi (*S.* Typhi) isolates which were resistant to ampicillin, chloramphenicol, and co-trimoxazole multidrug-resistant (MDR) per year.

Full antibiotic susceptibility results were available from 659 (72.2%) patient isolates, 239 (26.2%) had partial results, and 15 (1.7%) isolates had no susceptibility results because of a failure to regrow on subculture. The majority of isolates with data available (712/898, 79.3%) were susceptible to all antibiotics tested. There were 71/893 (8.0%) ampicillin-resistant, 70/865 (8.1%) chloramphenicol-resistant, and 67/885 (7.6%) co-trimoxazole–resistant isolates; 59/848 (7.0%) isolates with ampicillin, chloramphenicol, and co-trimoxazole results were MDR (see Table 2). Twelve (12/854, 1.4%) isolates were resistant to quinolones and 39/854 (4.6%) isolates were classified as being of intermediate susceptibility (Figure 6). The two isolates that were ciprofloxacin resistant were also nalidixic acid resistant. However, eight of the other nalidixic acid–resistant isolates had intermediate susceptibility to ciprofloxacin (two isolates were not tested against ciprofloxacin). Four MDR isolates also had intermediate susceptibility or resistance to quinolones, one of which also had intermediate susceptibility to ceftriaxone. In total, 778 isolates were re-subcultured in 2019 for testing against additional antibiotics or for confirmation of susceptibility results, of which 692 grew *S*. Typhi. Because of space, time, and cost constraints, not all isolates were retested against all antibiotics. However, 43/59 of the suspected MDR isolates grew on subculture, and all were confirmed as MDR. Results of quinolone susceptibility testing were confirmed for 25 isolates that had originally been classified as resistant or of intermediate susceptibility. On this basis, it was assumed that the original susceptibility results for isolates where retesting was not possible were correct. The majority of the MDR isolates came from an outbreak in Oudomxay Province in 2004 (37/59, 62.7% of recorded MDR cases). There have been no further MDR isolates reported from Oudomxay, and only four cases of MDR were recorded in the whole country since 2004 (Figure 7). The outbreak of MDR isolates in Oudomxay was probably clonal as all isolates had similar antibiotic susceptibility patterns. If the Oudomxay outbreak is excluded, the frequency of *S*. Typhi MDR was only 22/865 (2.5%). Of the 76 isolates recorded as resistant or intermediate to ceftriaxone, 73 (96%) were patients admitted between 2000 and 2004. There have been no isolates resistant or intermediate to ceftriaxone since 2009.

**Table 2:** Antibiotic susceptibility pattern for *Salmonella enterica* serovar Typhi isolates tested against seven antibiotics (*n* = total number of isolates tested against the particular antibiotic) over the 18 years

Antibiotic tested	Ampicillin (*n* = 893)			Azithromycin (*n* = 740)			Chloramphenicol (*n* = 865)			Ceftriaxone (*n* = 815)			Ciprofloxacin (*n* = 758)			Nalidixic acid (*n* = 819)			Co-trimoxazole (*n* = 885)			MDR
	S	I	R	S	I	R	S	I	R	S	I	R	S	I	R	S	I	R	S	I	R	
Number of isolates	818	4	71	739	0	1	788	7	70	739	65	11	723	33	2	798	9	12	814	4	67	59

S = susceptible; I= intermediate; R= resistant; MDR= multi-drug resistant.

### Environmental factors.

In the three provinces with highest typhoid inpatient incidence (Vientiane Capital, Vientiane Province, and Luang Namtha), the main drinking water source for villages changed from unprotected wells/bore water in 435/1,515 villages (28.7%) in 2005 to bottled water in 700/1,277 villages (54.8%) in 2015. There were significant decreases in people getting water from piped water, protected and unprotected wells, and rivers and streams (*P* < 0.005) between 2005 and 2015. The only water source that did not change markedly was water from mountain sources (*P* = 0.093) (www.decide.la).^17,19^ However, there were differences between provinces in the main water source for villages in 2015, with Vientiane Capital and Vientiane Province predominantly using bottled water (398/481 villages, 82.7% and 250/434 villages, 57.6%, respectively), whereas in Luang Namtha, majority of the people (258/362, 71.3%) sourced their water from mountain-fed water sources.

## DISCUSSION

These data from blood cultures received in a single hospital from diverse parts of Laos provide a minimal estimate of inpatient culture-positive typhoid incidence, with the most recent annual incidence in 2018 of 0.59 cases per 100,000 people. Clearly, this estimate of annual incidence of hospitalized typhoid is a significant underestimate of the true incidence of the disease in the community as few hospitals have blood culture facilities and not all typhoid patients will access hospitals or will be admitted. The Typhoid Global Burden of Disease (GBD) study from 2017 estimated 16,767 cases and 202 deaths from typhoid fever in Laos in 2017 through modeling, whereas we only identified 15 culture-confirmed inpatients in that year.^20^ The considerable difference is likely to be related to multiple factors, including incomplete national case capture in our study and the GBD estimates, in the absence of local sources of data on typhoid in Laos, being based on extrapolation of data from neighboring countries, which may not accurately reflect the incidence in Laos. There are important biases in the data from this study, including the fact that it did not homogenously cover the whole of the country, that the diagnosis was centralized in the capital with shipment of blood cultures from five provincial hospital sites, including one that was specifically sampled to investigate a typhoid outbreak. Majority of blood cultures (99%) were from inpatients, and hence, estimates will be heavily dependent on patient health-seeking behavior, access to provincial and central hospitals, and out-of-pocket cost of patient services, and therefore, our estimates are likely to be underestimates. Some outpatients were included, and the samples from the Oudomxay outbreak were mainly taken in the community. Access to healthcare centers and laboratories is difficult in many areas of Laos, particularly in isolated rural areas, which reduces the number of blood cultures taken and, therefore, currently makes accurate typhoid surveillance impossible. There is also very limited laboratory capacity in provincial hospitals, with only Mahosot Hospital providing an accessible, free blood culture service in the country. Although typhoid is a notifiable disease in Laos with the case definition mainly based on clinical diagnosis and not on culture-positive samples, without decentralized typhoid surveillance, accurate understanding of the epidemiology of typhoid in Laos will be imperfect. There is anecdotal evidence that the frequency of oral fluoroquinolone use in Laos has increased in communities and hospitals over the last 18 years. Hence, if patients with early symptoms of typhoid are being treated with such efficacious antityphoidal antibiotics at pharmacies and clinics, many patients with typhoid will not be admitted to hospital. We were unable to access data on fluoroquinolone consumption in Laos through time or the incidence of typhoid-associated complications, such as intestinal perforation. Nonetheless, our data are the only substantial body of data about laboratory-confirmed typhoid within Laos and, despite the shortcomings, enable some conclusions to be drawn about the national picture.

There has been a progressive drop in the annual number of typhoid patients detected since 2010, with *S*. Typhi going from being the most common organism in blood cultures in the early 2000s to being recently replaced by *Escherichia coli (E. coli)* (unpublished data, Mahosot Hospital). The reason for this decrease in in-hospital incidence is unclear. Although typhoid immunization is not part of the routine vaccination program in Laos, one potential explanation is that people increasingly have access to safer water sources. From the Lao census data collected in 2005 and 2015, there has been a significant change in the sources of drinking water for many people in Laos, with people in Vientiane Capital and Vientiane Province changing from using unprotected well and borehole water in 2005 to predominantly getting their water from bottles in 2015. Nevertheless, there is still a high percentage of the Lao population at risk of exposure to contaminated water sources both in rural and urban settings. One million people (16.7%) still do not have access to basic water supply, and open defecation remains common, with almost 24% of the population defecating in the open, with the proportion being higher among rural than urban populations (32.6% compared with 4.3%).^21,22^ However, what effect these changes in sources of drinking water have had on typhoid transmission in Laos has not been properly studied.

The incidence of the disease appears to be highest in the rural north of the country, such as in Luang Namtha, bordering China, where fever surveillance studies have been undertaken since 2008.^18^ Although the incidence of typhoid in China is reported to be low, higher incidences are reported from the southern provinces that border Laos and Vietnam.^23^ The one site in southern Laos, at Salavan Provincial Hospital, had a comparatively low typhoid inpatient incidence. This province borders neighboring provinces in Vietnam and Thailand, where low typhoid incidence has also been reported.^3,4^ In Vietnam, most patients present in the Mekong Delta area in the far south, and in Thailand, most cases are seen in the northwest.^3,4,24^

The highest number of typhoid cases in Vientiane capital came from a rural district (Sangthong), which is the furthest district from the city center. The high number of *S*. Typhi cases in rural populations has also been seen in other studies in the region, with the highest burden of typhoid fever in infants seen in rural communities in Cambodia.^25^ With the difficulties of access to immunization in rural Laos, improving penetration of vaccines into the hinterland will be vital for *S.* Typhi control. Given the continuing widespread endemicity of typhoid in Laos, we agree with United Kingdom recommendations that most travelers to Laos should be offered typhoid vaccine, especially because many of these are likely to be more adventurous than most travelers to other countries.^16^

Although a high proportion of antimicrobial resistance have been reported in neighboring countries, MDR *S*. Typhi frequency has been very low in Laos over the last 18 years and also appears to have recently declined in the region. Although a high frequency of MDR *S*. Typhi in children was reported from Siem Reap, Cambodia, all haplotype H58 and of predominantly ciprofloxacin intermediate susceptibility,^26^ another study from Phnom Penh reported a decline in MDR cases over a 7-year period.^27^ This decline in MDR frequency has also been seen in Vietnam since the first-line treatment shifted from ampicillin, chloramphenicol, and co-trimoxazole to cephalosporins and quinolones. *Salmonella enterica* serovar Typhi antibiotic resistance in Thailand also remains uncommon.^3,4^ Of the 59 Lao MDR isolates, the majority of them (93.2%) were isolated between 2000 and 2004 with only four isolates cultured since 2004; the majority came from a single, probably clonal, outbreak in Oudomxay Province. The majority (96%) of ceftriaxone intermediate and resistant isolates were also obtained between 2000 and 2004. What led to this dramatic fall in the prevalence of MDR and reduced ceftriaxone susceptibility after 2004 is unknown. Analysis of a subset of 183 Lao *S*. Typhi isolates identified 10 circulating genotypes.^28^ Most MDR isolates were from the widely spread genotype 4.3.1, with two MDR isolates of genotype 3.2.1. Genotype 3.2.1 appears to be of Asian origin, and MDR isolates within this genotype have been found in Thailand and Vietnam with antimicrobial resistant (AMR) genes carried on plasmids of the IncHI1 plasmid sequence type 2.^29,30^

The reason for this comparative rarity of MDR and reduced fluoroquinolone and ceftriaxone susceptibility in Laos, despite the widespread movement of people and widespread and uncontrolled antibiotic use,^31,32^ is unclear. Without objective data on temporal and spatial variation in key antibiotic use in Laos, it is hard to judge the relative importance or otherwise of inappropriate antibiotic use as AMR drivers.

The clinical trial of the treatment of uncomplicated typhoid conducted at Mahosot Hospital in 2001–2003 demonstrated that 3 days of ofloxacin was more efficacious than 14 days of chloramphenicol.^13^ Although there have been no subsequent clinical trials, the current clinical evidence and the rarity of fluoroquinolone resistance support the continued use of 3 days of ofloxacin as the first-line treatment for suspected typhoid in Laos.

Apart from the outbreak years of 2002 and 2004, when most cases were seen in March, there is generally a peak in cases in May and June at the end of the dry season followed by reduction of cases in the mid-wet season. This pattern was also described in Cambodia, with most patients presenting in the hot dry months of March to May.^27^ Other studies in Asia, such as in neighboring Thailand, have shown a more seasonal pattern following wet seasons and flooding.^3,33^ The factors driving this earlier occurrence of cases in Laos could include limited water sources toward the end of the dry season, with people having to look for other sources.

In conclusion, this is the largest epidemiological report on typhoid in Laos. To inform decisions about vaccine introduction, more typhoid surveillance data and cost-effectiveness analysis are needed. As the use of fluoroquinolones to treat typhoid is a national policy, and they can be accessed in central, district, and provincial hospitals as well as pharmacies and private clinics, continued surveillance of *S*. Typhi susceptibility to these agents is critical. Greater understanding of the drivers of fluoroquinolone typhoid resistance could inform interventions in Laos to reduce the risk of a worsening situation. Although multidrug resistance and fluoroquinolone resistance are not currently major public health problems, continuous surveillance and antibiotic stewardship are necessary to prevent a rise in numbers.

## Supplemental figures

Supplemental materials
