# Supplementary material for: Typhoid in Laos: An 18-Year Perspective
Source: Am J Trop Med Hyg. 2020 Jan 27;102(4):749. doi: 10.4269/ajtmh.19-0637 (PMC7124924; doi:10.4269/ajtmh.19-0637)
Supplement: Supplementary file 1 [file tpmd190637.SD1.pdf]

The following are supplemental materials and will be published online only

Supplementary material

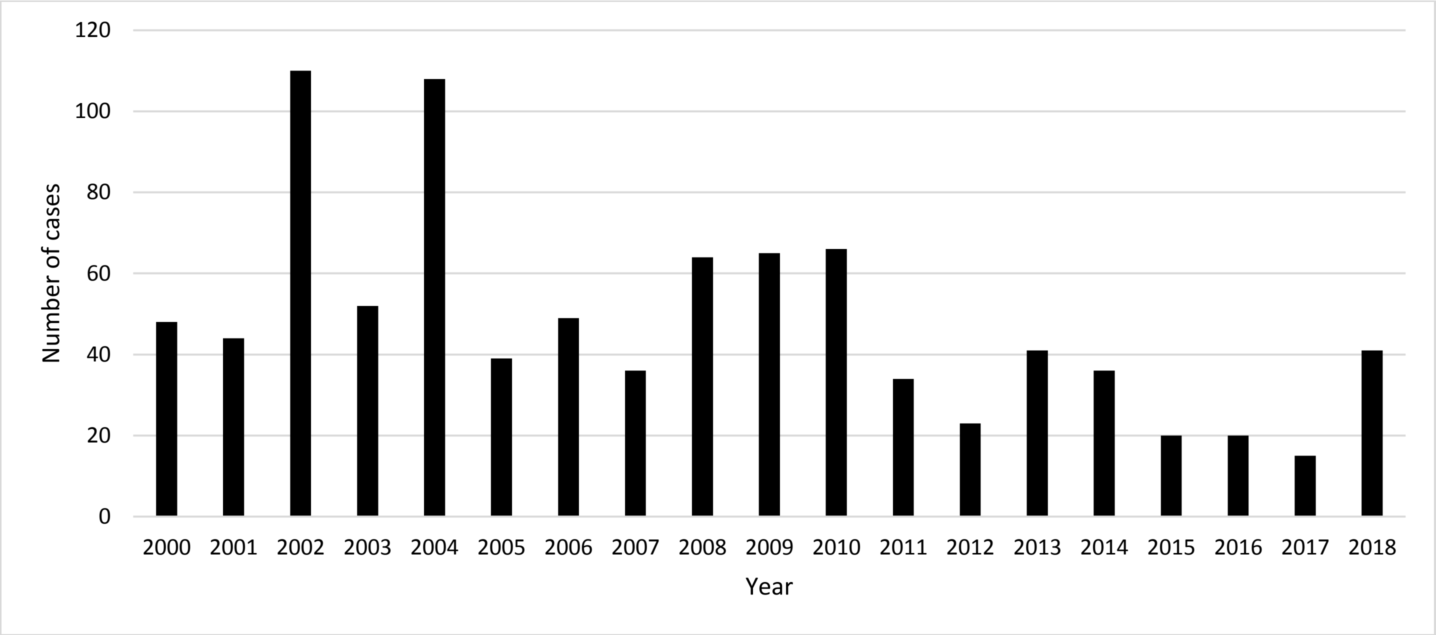

Supplementary Figure 1. Total number of inpatients recorded with *S.* Typhi blood stream infections per year

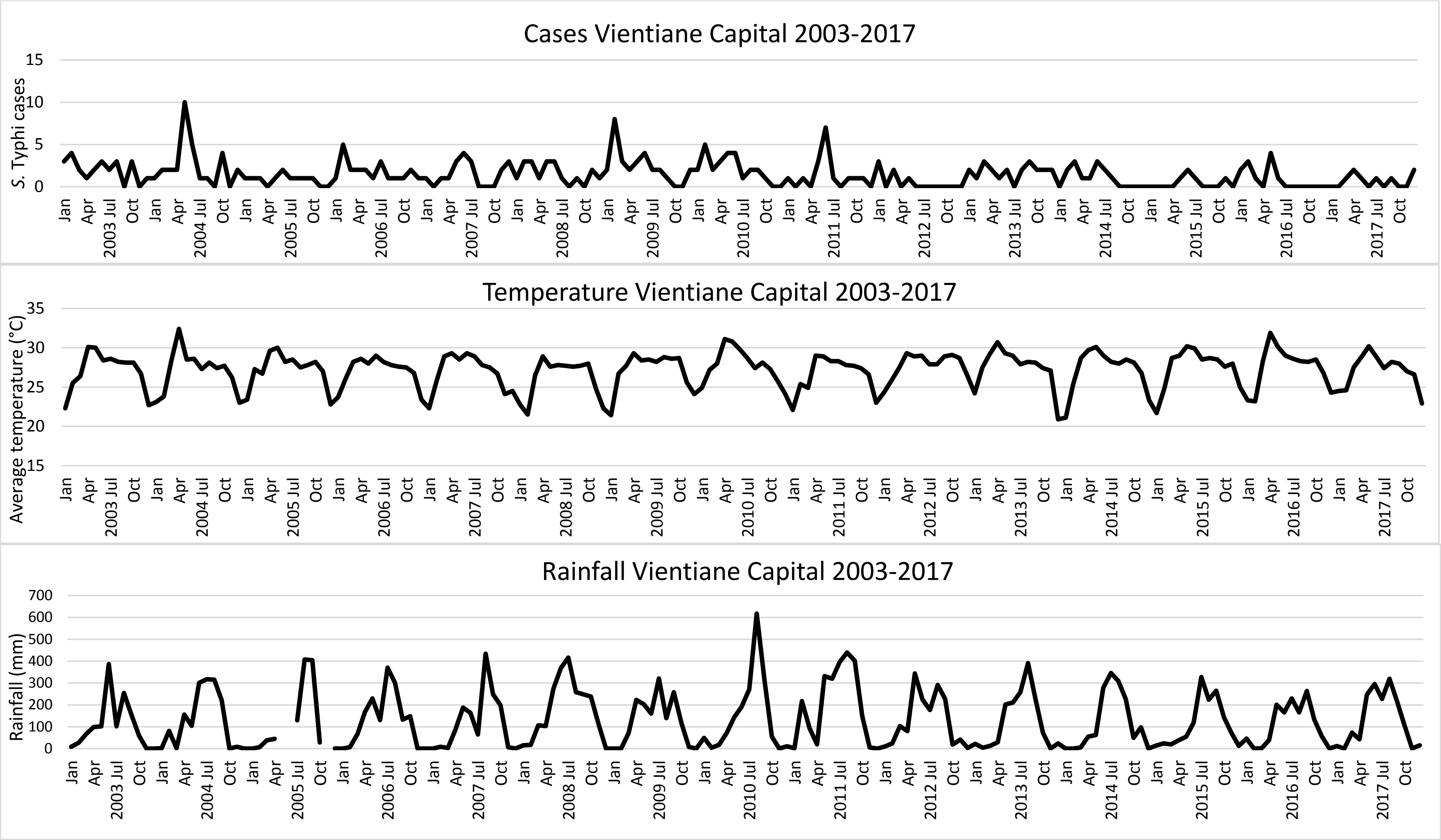

Supplementary Figure 2. Number of inpatients recorded with *S.* Typhi per month, mean temperature and mean rainfall for Vientiane Capital

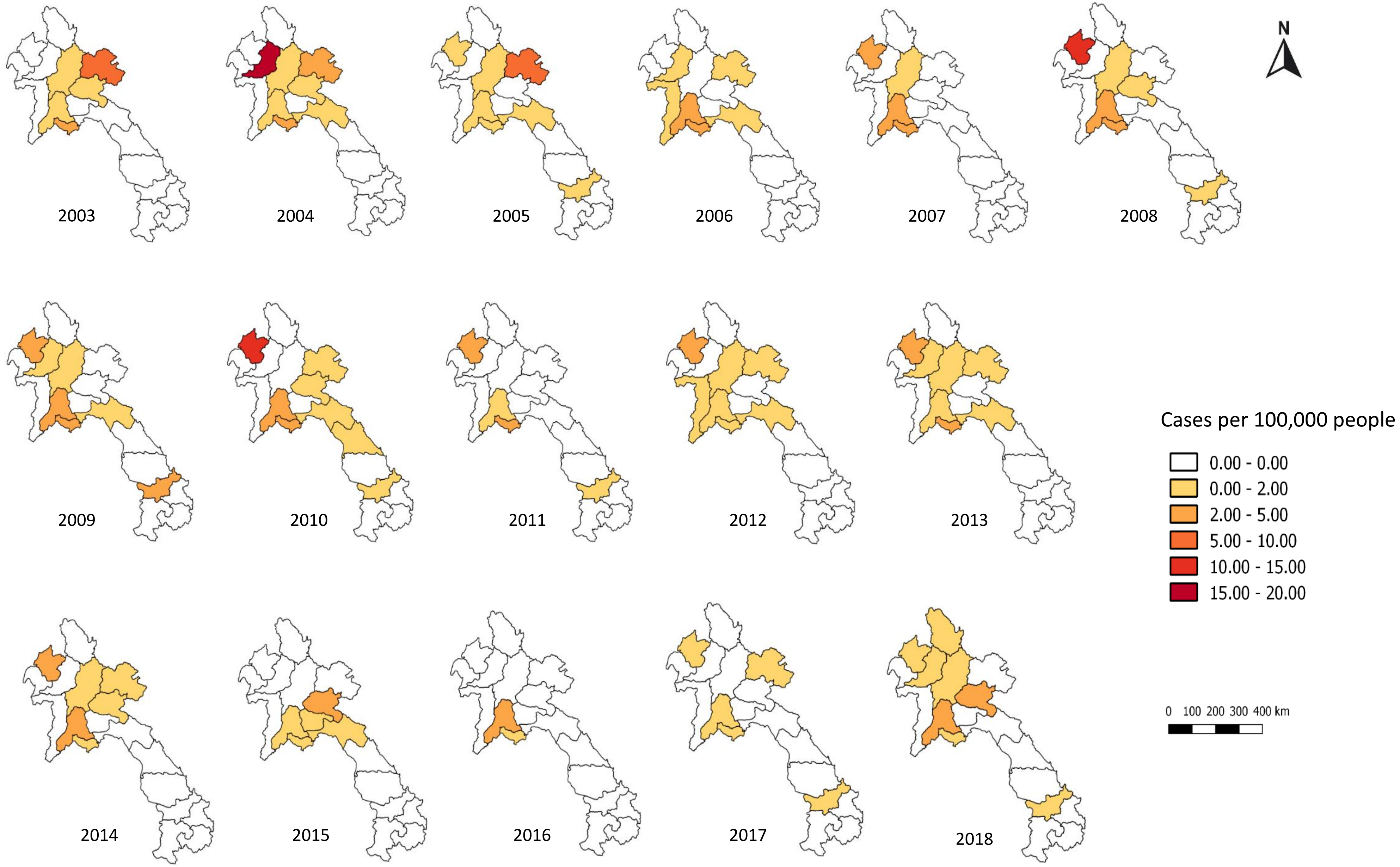

Supplementary Figure 3. *S.* Typhi cases diagnosed in hospital per 100,000 people in the community by home province by year

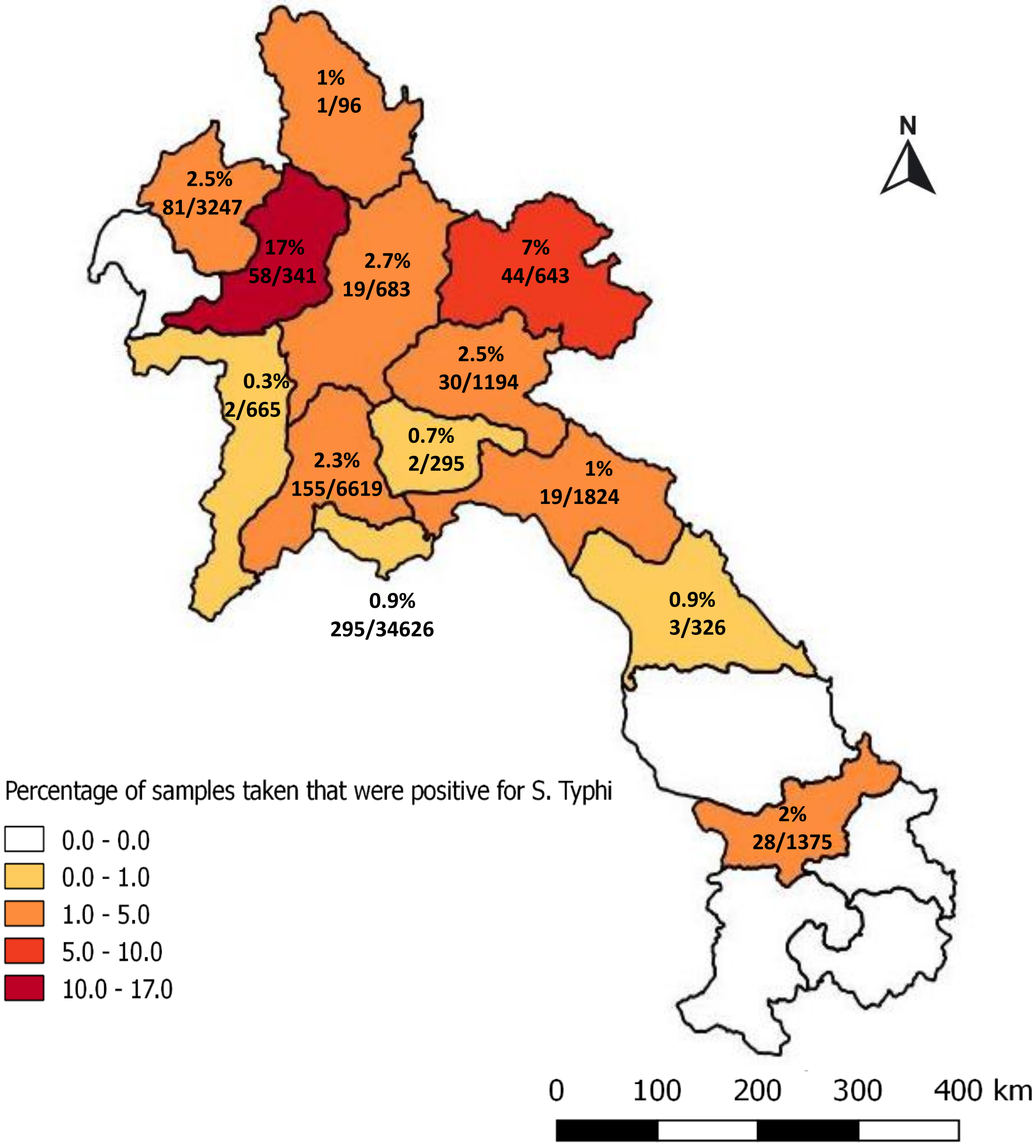

Supplementary Figure 4. Percentage of all samples taken that were positive for *S.* Typhi per province between 2000- 2018. Highest percent of positive cases came from Oudomxay Province which reflects the 2002 outbreak

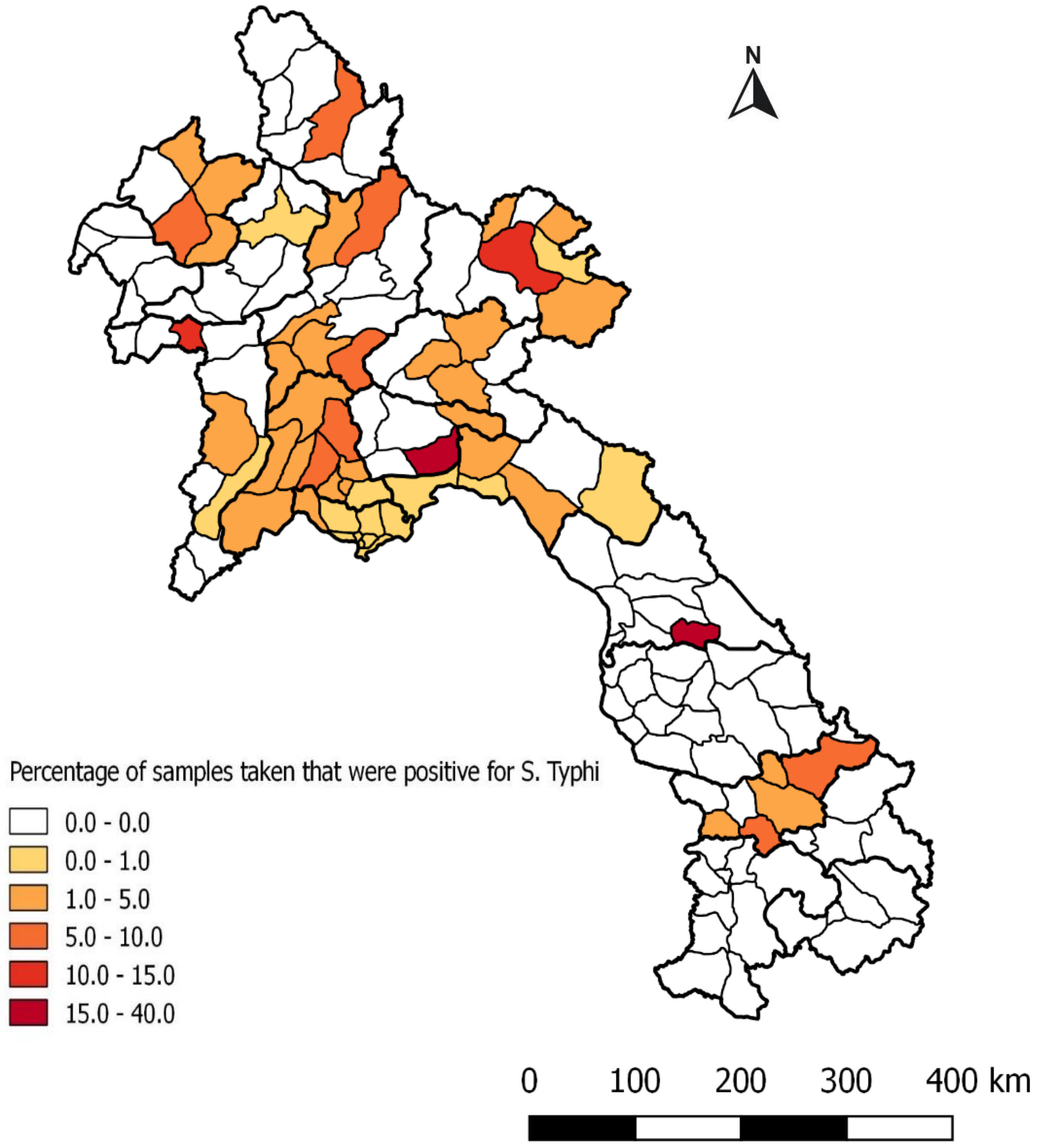

Supplementary Figure 5. Percentage of all blood culture samples taken that were positive for *S.* Typhi per community home district population between 2000- 2018.
